# Prognostic Performance of Cystatin C in COVID-19: A Systematic Review and Meta-Analysis

**DOI:** 10.3390/ijerph192114607

**Published:** 2022-11-07

**Authors:** Michal Matuszewski, Yurii Reznikov, Michal Pruc, Frank W. Peacock, Alla Navolokina, Raúl Júarez-Vela, Lukasz Jankowski, Zubaid Rafique, Lukasz Szarpak

**Affiliations:** 1 Department of Anaesthesiology and Intensive Therapy, Central Clinical Hospital of the Ministry of Interior and Administration, 02-507 Warsaw, Poland; 2 European School of Medicine, International European University, 03187 Kyiv, Ukraine; 3 Research Unit, Polish Society of Disaster Medicine, 05-806 Warsaw, Poland; 4 Henry JN Taub Department of Emergency Medicine, Baylor College of Medicine Houston, Houston, TX 77030, USA; 5 GRUPAC, Department in Nursing, University of La Rioja, 26004 Logroño, Spain; 6 Clinic of Transplantation Medicine, Nephrology and Internal Diseases, Medical University of Warsaw, 02-097 Warsaw, Poland; 7 Institute of Outcomes Research, Maria Sklodowska-Curie Medical Academy, 00-136 Warsaw, Poland

**Keywords:** Cystatin C, cystatin 3, SARS-CoV-2, novel coronavirus, COVID-19, severity

## Abstract

Cystatin C is a specific biomarker of kidney function. We perform this meta-analysis to determine the association of Cystatin C with the COVID-19 severity. In this systematic review and meta-analysis, we searched PubMed, EMBASE, Cochrane library, and Web of Science for studies published until 2nd September 2022 that reported associations between Cystatin C levels and COVID-19 severity. The analysis was performed using a random-effects model to calculate pooled standard mean difference (SMD). Twenty-five studies were included in the meta-analysis. Pooled analysis showed statistically significant differences of Cystatin C levels among survive vs. decreased patients (0.998 ± 0.225 vs. 1.328 ± 0.475 mg/dL, respectively; SMD = −2.14; 95%CI: −3.28 to −1.01; *p* < 0.001). Cystatin C levels in COVID-19 severe vs. non-severe groups varied and amounted to 1.485 ± 1.191 vs. 1.014 ± 0.601 mg/dL, respectively (SMD = 1.81; 95%CI: 1.29 to 2.32; *p* < 0.001). Additionally, pooled analysis showed that Cystatin C levels in patients with acute kidney injury (AKI) was 1.562 ± 0.885 mg/dL, compared to 0.811 ± 0.108 mg/dL for patients without AKI (SMD = 4.56; 95%CI: 0.27 to 8.85; *p* = 0.04). Summing up, Cystatin C is a potentially very good marker to be used in the context of COVID-19 disease due to the prognosis of patients’ serious condition, risk of AKI and mortality. In addition, Cystatin C could be used as a marker of renal complications in COVID-19 other than AKI due to the need to monitor patients even longer after leaving the hospital.

## 1. Introduction

Global healthcare systems have been under intense pressure since the coronavirus disease 2019 (COVID-19) caused by severe acute respiratory syndrome coronavirus-2 (SARS-CoV-2) was announced in China in late 2019 and spread around the world [1]. Acute respiratory distress syndrome (ARDS) and mortality are the most severe signs of symptomatic COVID-19, but moderate fever (>37.5 °C) and cough are also common, and the disease has an erratic course. Due to this diversity, there is an urgent need for disease severity biomarkers so that patients may be managed effectively, and deadly consequences can be avoided. The use of biomarkers in diagnosis, risk assessment, and medical decision-making is common. Mortality has been linked to markers of organ failure, coagulation, and inflammation in COVID-19 hospitalized patients [2,3]. Identifying which patients are likely to pass away can help with early therapy intensification and closer monitoring. Additionally, research into novel biomarkers may provide fresh insights into the pathogenesis of COVID-19 and its consequences.

All nucleated cells generate Cystatin C, a small molecule protein that is a member of the cysteine protease inhibitor superfamily. Cystatin C is a nonglycosylated basic protein that is continuously generated by nucleated cells and is easily filtrable from the glomerulus. The proximal tubular cells totally catabolize Cystatin C, preventing its return to the circulatory system [4]. Cystatin C levels can more accurately reflect changes in glomerular filtration rate (GFR) than serum creatinine does. Unlike the traditional detection indices, Cystatin C is unaffected by age, sex, race, infection, liver illness, or inflammation [5]. As a result, it is frequently utilized in the diagnosis and assessment of renal disorders [6]. It has been demonstrated that Cystatin C has higher sensitivity to changes in borderline renal function and rises earlier than creatinine in a variety of patient populations, including diabetic, surgical, and cardiovascular patients [7,8,9]. Moreover, a growing number of studies have shown conclusively that Cystatin C contributes to the pathophysiology of the immunomodulatory responses seen in inflammatory conditions and infections. Cystatin C can control the release of a variety of cytokines, including nitric oxide, interleukin-12, interleukin-10, and tumor necrosis factor [10]. The inducible isoform of NO synthase (iNOS), in particular, is activated by Cystatin C and is principally in charge of the excessive NO synthesis seen in local and systemic proinflammatory conditions [11]. As a result, extremely reactive NO derivatives are produced, nitrosative stress is caused, and various intracellular components undergo irreversible changes with consequent cell apoptosis and organ failure occurring. While elevated blood levels of Cystatin C in individuals with COVID-19 are likely to indicate the existence of renal impairment, such as acute kidney injury (AKI), they may also be a sign of the excessive systemic inflammatory and pro-oxidant state that distinguishes patients with COVID-19 [12]. Cystatin C also may be employed as a trustworthy biological marker to predict AKI, particularly in assisting with early clinical identification of AKI, and AKI in hospitalized patients with COVID-19 is as high as 43% [13,14]. Renal biomarkers may help with early risk classification, monitoring, and therapy in patients with COVID-19 given the more common development of renal impairment in this population [14]. Nevertheless, the availability of biomarkers that might indicate not just the early presence of renal failure but also other aberrant processes, such as systemic inflammation, oxidative stress, and cytokine storm, may be particularly helpful in the COVID-19 pandemic. For these reasons, we decided to conduct a review and meta-analysis on the prognostic role of Cystatin C and its predictive role regarding the occurrence of AKI in patients with COVID-19.

## 2. Materials and Methods

This systematic review and meta-analysis was performed in accordance to the Preferred Reporting Items for Systematic Reviews and Meta-Analyses (PRISMA) Statement [15]. Before commencing the study, all authors agreed on the analysis methods and the inclusion and exclusion criteria to be used.

### 2.1. Study Identification

We searched PubMed, EMBASE, Cochrane library, and Web of Science. An initial search was performed on 21 May 2020, including publications from 1st January 2020, followed by a final search on 2 September 2022 which incorporated all the manuscripts up to this date. The search was conducted using the terms: “Cystatin C” OR “cystatin 3” AND “SARS-CoV-2” OR “COVID-19” OR “novel coronavirus”. Additionally, references of relevant reviews, full-text articles, and the included literature were screened for additional studies that may have been missed. All references were saved in an EndNote (End Note, Inc., Philadelphia, PA, USA) library used to identify the duplicates.

### 2.2. Study Selection Criteria

Two reviewers independently screened articles according to pre-specified inclusion and exclusion criteria. The inclusion criteria were studies reporting Cystatin C levels among patients with COVID-19 who survive vs. dead or severe vs. non-severe COVID-19 status. Additionally, we also include trials reporting Cystatin C levels among patients with COVID-19 with and without acute kidney injury.

Furthermore, this review excluded the following types of studies: (1) papers not containing comparator group; (2) paper referring to pediatric population; (3) conference or poster papers; (4) reviews or meta-analyses; (5) case reports; (6) articles not containing original data; (7) articles published in other than English language.

### 2.3. Data Extraction

Two independent authors (M.M. and M.P.) extracted the data using predefined extraction form: first author surname, publication year, study designs, country of publication, study population characteristics, Cystatin C levels, and study quality. Disagreements were resolved by the third author (L.S.).

### 2.4. Quality Assessment

We referred to the Cochrane Handbook to guide the synthesis [16]. The methodological quality of the observational studies was assessed using the Newcastle Ottawa Scale [17]. NOS measures the quality of a study based on three aspects: selection, comparability, and exposure. The maximum scores of these three aspects were 4, 2 and 3 stars, respectively. Studies with NOS scores ≥ 7 were considered to be high-quality studies.

### 2.5. Statistical Analysis

Statistical analysis was performed using RevMan (ver. 5.4, The Nordic Cochrane Centre, The Cochrane Collaboration, Copenhagen, Denmark). The standard mean difference (SMD) was calculated with 95% confidence intervals (CIs). The SMD was calculated by using random-effects models. When the continuous outcome was reported in a study as median, range, and interquartile range, we estimated means and standard deviations using the Hozo et al. formula [18]. Heterogeneity was assessed using the tau coefficient and measured using the I2 index and we consider percentages of around I2 = 25%, I2 = 50%, and I2 = 75% as low, medium, and high heterogeneity, respectively [19]. The *p*-value ≤ 0.05 cut-point was used to declare statistical significance. Potential publication bias was assessed using funnel plots, and where possible, Egger’s regression test was performed. However, when a limited number of studies (<10) were included in the analysis, publication bias was not evaluated.

## 3. Results

### 3.1. Study Selection and Characteristics

**Figure 1:**
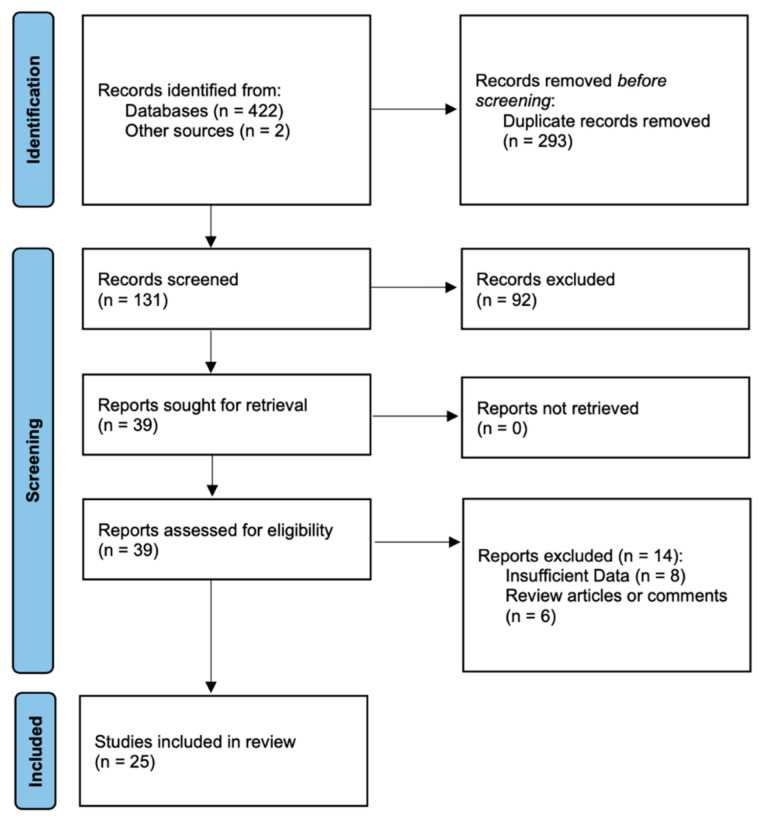
The flow diagram for study search process.

Overall, 422 studies were identified through database searches, and two additional articles were retrieved from the bibliographies of the included studies. Two hundred ninety-three duplicates were removed, and the remaining 131 articles were screened by title and abstract, which resulted in the exclusion of 92 irrelevant articles. Full-text screening was performed on 39 studies, and data for 25 studies were extracted for this meta-analysis [20,21,22,23,24,25,26,27,28,29,30,31,32,33,34,35,36,37,38,39,40,41,42,43,44]. Figure 1 depicts the various exclusions and selection procedures.

The systematic review included articles published between 2020 and 2022, comprising a total of 3916 COVID-19 participants (Table 1). The final set considered of 25 eligible studies [20,21,22,23,24,25,26,27,28,29,30,31,32,33,34,35,36,37,38,39,40,41,42,43,44,45,46], including 18 articles conducted in China [21,22,23,25,26,27,28,31,33,34,36,37,38,39,41,42,43,44], two articles in Turkey [32,40], and one article each from the following countries: Iraq [20], India [24], Mexico [29], USA [30] and Egypt [35].

### 3.2. Meta-Analysis

**Figure 2:**
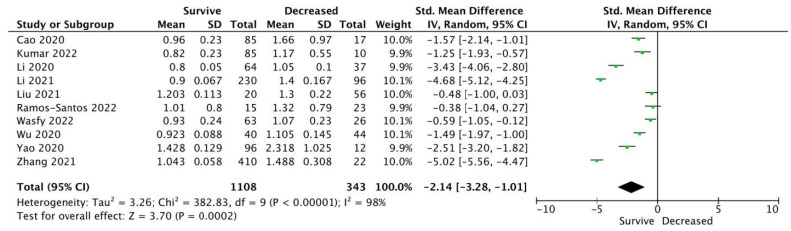
Forest plot of Cystatin C levels among COVID-19 survive vs. decrease patients. The center of each square represents the standard mean differences for individual trials, and the corresponding horizontal line stands for a 95% confidence interval. The diamonds represent pooled results. Legend: CI: confidence interval; SD: standard deviation [21,24,25,26,28,29,35,36,39,42].

Ten studies reported Cystatin C levels among patients who survived to hospital discharge vs. patients who decreased. Pooled analysis showed statistically significantly differences between those groups (0.998 ± 0.225 vs. 1.328 ± 0.475 mg/dL, respectively; SMD = −2.14; 95%CI: −3.28 to −1.01; *p* < 0.001; Figure 2).

**Figure 3:**
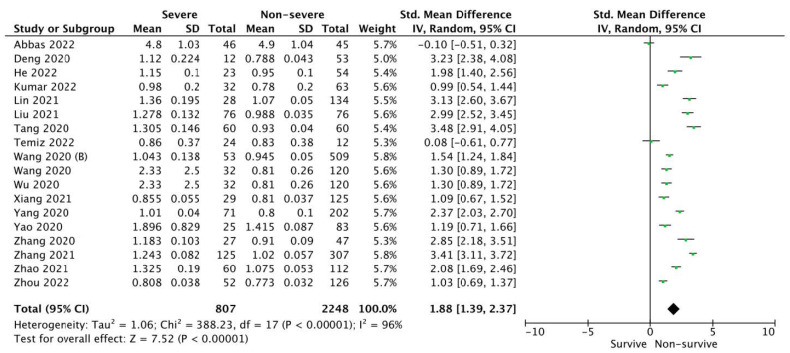
Forest plot of Cystatin C levels among severe vs. non-severe patients with COVID-19. The center of each square represents the standard mean differences for individual trials, and the corresponding horizontal line stands for a 95% confidence interval. The diamonds represent pooled results. Legend: CI: confidence interval; SD: standard deviation [20,22,23,24,27,28,31,32,33,34,36,37,38,39,41,42,43,44].

Pooled analysis of Cystatin C levels in COVID-19 severe vs. non-severe groups varied and amounted to 1.485 ± 1.191 vs. 1.014 ± 0.601 mg/dL, respectively (SMD = 1.81; 95%CI: 1.29 to 2.32; *p* < 0.001; Figure 3).

**Figure 4:**
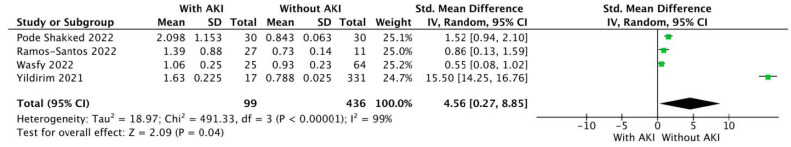
Forest plot of Cystatin C levels among Patients with COVID-19 with and without acute kidney injury (AKI). The center of each square represents the standard mean differences for individual trials, and the corresponding horizontal line stands for a 95% confidence interval. The diamonds represent pooled results. Legend: AKI: acute kidney injury; CI: confidence interval; SD: standard deviation [29,30,35,40].

Additionally, three studies reported Cystatin C levels among patients with COVID-19 with and without AKI. Pooled analysis showed that Cystatin C levels in patients with AKI was 1.562 ± 0.885 mg/dL, compared to 0.811 ± 0.108 mg/dL for patients without AKI (SMD = 4.56; 95%CI: 0.27 to 8.85; *p* = 0.04; Figure 4).

## 4. Discussion

Due to the limited medical resources available during the COVID-19 pandemic, the early and rapid evaluation of severe patients with COVID-19 is essential to ensuring early medical surveillance and therapies for these patients. This meta-analysis evaluated the serum Cystatin C levels of patients with COVID-19. Although some studies have investigated the association between Cystatin C and AKI. Moreover, meta-analysis by Zinellu et al. evaluate the relation between Cystatin C and COVID-19, however, contain only 13 articles [45]. In this meta-analysis, twenty-five studies were included and analyzed which makes it the broadest and most comprehensive meta-analysis available in this area. The results of our meta-analysis show the potential clinical role of Cystatin C in predicting severe COVID-19 in patients. Serum Cystatin C concentrations in particular were linked to COVID-19 mortality [21,24,26,27,28,29,35,36,39,42], severity [20,22,23,24,27,28,31,32,33,34,36,37,38,39,41,42,43,44], and the onset of acute kidney injury [29,30,35,40].

Although the co-expression of ACE2 receptors and transmembrane serine proteases (TMPRSSs) is essential for SARS-CoV-2 entrance into host cells, the exact mechanism causing kidney damage after COVID-19 infection is yet unknown [46]. Podocytes and proximal straight tubule cells have relatively substantial co-expression of the genes ACE2 and TMPRSS, according to a single-cell transcriptome investigation [47]. Consequently, a direct viral infection may be the cause of kidney damage. Furthermore, kidney cell injury might result from cytokine storm syndrome, which is connected to sepsis after SARS-CoV-2 infection [48]. Additionally, volume loss and multiple organ failure may cause renal impairment. Since glomerular transport accounts for the majority of Cystatin C excretion, a decrease in glomerular filtration rate would coincide with an increase in Cystatin C levels. The elevated amounts of Cystatin C may be explained by a reduction in the functional pores’ pore widths, according to previous research [49]. The term “Shrunken pore syndrome” was recently developed to indicate a smaller pore size of the glomerular membranes, which may explain why Cystatin C is a better predictor of death [50]. According to our research, severe SARS-CoV-2 infection may cause kidney injury since Cystatin C levels were considerably higher in patients with severe COVID-19 illness than in patients with non-severe COVID-19.

Our study also shows the important role of Cystatin C in the prediction of AKI among patients with COVID-19—AKI vs. non-AKI (SMD = 6.28; 95%CI: 1.11 to 11.44; *p* = 0.02) Moreover, COVID-19 increases CKD risk in addition to AKI related to it, according to American research that used electronic health data from the Veterans Health Administration to carry out a thorough evaluation long-COVID-19. Among those who experienced a major illness, this risk was the highest. Even after the initial 30 days following COVID-19 diagnosis, urinary tract infections, AKI, and CKD have been associated with poor renal symptoms in patients who needed to be hospitalized [51]. Patients with COVID-19 in China showed that 35% of patients had renal impairment (estimated glomerular filtration rate [eGFR] 90 mL/min/1.73 m^2^) 6 months following hospitalization for COVID-19. Surprisingly, 13% of patients who did not experience AKI while hospitalized exhibited a decline in eGFR during the follow-up [52]. A 30% loss in renal function was seen in around 5% of the study’s more than 1.7 million participants, 90,000 of whom are COVID-19 survivors with symptoms lasting at least 30 days (eGFR). This eGFR decreased by 30% more frequently in long-COVID-19 infected individuals than in healthy controls, or 25% more frequently in those who survived more severe illness. However, many people who were not hospitalized were nonetheless impacted by the disease [53]. When we are dealing with a significant number of patients with renal diseases and some studies indicate that 5% of vaccinated individuals require a long time to acquire COVID-19 while 11% of the unvaccinated group may face substantial nephrological challenges both during and after the pandemic occurs [54]. Cystatin C and its predictive function in terms of kidneys could also be used to predict other kidney diseases related to COVID-19 apart from AKI, although AKI as the most serious and fastest progressing one seems to be the most important–nevertheless, the ability to quickly test for levels Cystatin C among patients who have already left the hospital additionally emphasizes the role of this marker in terms of delayed complications of the disease [55].

These findings collectively lend credence to the idea that elevated serum Cystatin C levels in severe COVID-19 may be caused by one or more coexisting processes, such as impaired renal function, excessive proinflammatory cytokine release, antiviral effects, iNOS-mediated stimulation of NO synthesis, and cytokine storm. This meta-analysis also shows the potential use of Cystatin C as a prognostic marker of patients with COVID-19 and the early diagnosis of AKI and other subsequent renal disorders.

## 5. Conclusions

Cystatin C is a potentially very good marker to be used in the context of COVID-19 disease due to the prognosis of patients’ serious condition, risk of AKI and mortality. In addition, Cystatin C could be used as a marker of renal complications in COVID-19 other than AKI, due to the need to monitor patients even longer after leaving the hospital.

## Figures and Tables

**Table 1:** Baseline characteristics of the included studies.

Study	Country	Study Group	No. of Patients	Age	Sex, Male	Cystatin C Level	NOS Scale
Abbas et al., 2022 [20]	Iraq	Non-severe	35	56.82 ± 12.574	28 (80.0%)	0.49 ± 0.104	8
		Severe	36	63.04 ± 11.143	29 (80.6%)	0.48 ± 0.103	
Cao et al., 2020 [21]	China	Survivors	85	54.75 ± 3.17	40 (47.1%)	0.96 ± 0.23	9
		Non-survivors	17	72 ± 4.5	13 (76.5%)	1.66 ± 0.97	
Deng et al., 2020 [22]	China	Non-severe	53	34 ± 2	24 (45.3%)	1.12 ± 0.224	8
		Severe	12	33.25 ± 2	12 (100%)	0.788 ± 0.043	
He et al., 2022 [23]	China	Non-severe	54	49.7 ± 5.3	26 (48.1%)	1.15 ± 0.1	8
		Severe	23	63.25 ± 2.25	12 (52.2%)	0.95 ± 0.1	
Kumar et al., 2022 [24]	India	Non-severe	63	52.1 ± 11.1	NS	0.82 ± 0.23	7
		Severe	32	54.8	NS	1.17 ± 0.55	
Li et al., 2020 [25]	China	Survivors	64	54.09 ± 14.95	30 (46.9%)	0.8 ± 0.05	8
		Non-survivors	37	71.76 ± 10.012	23 (62.2%)	1.05 ± 0.1	
Li et al., 2021 [26]	China	Survivors	230	56.25 ± 3.83	108 (47.0%)	0.9 ± 0.067	8
		Non-survivors	96	70.75 ± 3	63 (65.6%)	1.4 ± 0.167	
Lin et al., 2021 [27]	China	Non-severe	134	59.8 ± 13.0	64 (47.8%)	1.36 ± 0.195	8
		Severe	28	70.1 ± 12.7	20 (71.4%)	1.07 ± 0.05	
Liu et al., 2021 [28]	China	Non-severe	76	62.9 ± 9.3	49 (64.5%)	1.203 ± 0.113	9
		Severe	76	64.5 ± 9.3	49 (64.5%)	1.3 ± 0.22	
Ramos-Santos et al., 2022 [29]	Mexico	Without AKI	11	60.2 ± 10.2	7 (63.6%)	0.73 ± 0.14	9
		With AKI	27	52.5 ± 14.9	21 (77.8%)	1.39 ± 0.88	
		Survivors	15	NS	NS	1.01 ± 0.80	
		Non-survivors	23	NS	NS	1.32 ± 0.79	
Pode Shakked et al., 2022 [30]	USA	Without AKI	30	65.58 ± 2.93	14 (63.6%)	0.843 ± 0.063	8
		With AKI	22	44.7 ± 3.7	17 (56.7%)	2.098 ± 1.153	
Tang et al., 2020 [31]	China	Non-severe	60	54.25 ± 4.75	26 (43.3%)	1.305 ± 0.146	8
		Severe	60	62.98 ± 6	40 (66.7%)	0.93 ± 0.04	
Temiz et al., 2022 [32]	Turkey	Non-severe	24	53.96 ± 15.4	NS	0.86 ± 0.37	7
		Severe	12	71.42 ± 14.62	NS	1.52 ± 0.66	
Wang et al., 2020 [33]	China	Non-severe	35	38.5 ± 11.5	17 (48.6%)	2.33 ± 2.5	8
		Severe	10	44 ± 9.8	6 (60.0%)	0.81 ± 0.26	
Wang et al., 2020 (B) [34]	China	Non-severe	509	47.5 ± 5.3	164 (32.2%)	1.043 ± 0.138	8
		Severe	53	57.75 ± 4.25	7 (13.2%)	0.945 ± 0.05	
Wasfy et al., 2022 [35]	Egypt	Without AKI	64	60 ± 2	37 (57.8%)	0.93 ± 0.23	9
		With AKI	25	65.5 ± 2.5	14 (56.0%)	1.06 ± 02.5	
		Survivors	63	NS	NS	0.93 ± 0.24	
		Non-survivors	26	NS	NS	1.07 ± 0.23	
Wu et al., 2020 [36]	China	Survivors	40	49.28 ± 4.1	31 (77.5%)	0.934 ± 0.088	9
		Non-survivors	44	67.8 ± 3.9	29 (65.9%)	1.105 ± 0.145	
Xiang et al., 2021 [37]	China	Non-severe	125	NS	NS	0.855 ± 0.055	7
		Severe	29	NS	NS	0.81 ± 0.037	
Yang et al., 2020 [38]	China	Non-severe	202	47.6 ± 1.1	101 (50.0%)	1.01 ± 0.04	7
		Severe	71	53.5 ± 1.9	33 (46.5%)	0.8 ± 0.1	
Yao et al., 2020 [39]	China	Non-severe	83	47.5 ± 3.67	30 (36.1%)	1.896 ± 0.829	9
		Severe	25	59.9 ± 6.28	13 (52.0%)	1.415 ± 0.087	
		Survivors	96	48.72 ± 4.79	7 (58.3%)	1.428 ± 0.129	
		Non-survivors	12	63.6 ± 6.5	3 (25.0%)	2.318 ± 1.025	
Yildirim et al., 2021 [40]	Turkey	Without AKI	331	37 ± 2.67	146 (44.1%)	0.788 ± 0.025	8
		With AKI	17	71.6 ± 2.6	12 (70.6%)	1.63 ± 0.225	
Zhang et al., 2020 [41]	China	Non-severe	47	60.8 ± 3.3	18 (39.3%)	1.183 ± 0.103	8
		Severe	27	70.8 ± 5.8	18 (66.7%)	0.91 ± 0.09	
Zhang et al., 2021 [42]	China	Survivors	410	52.5 ± 4.7	219 (53.4%)	1.043 ± 0.058	9
		Non-survivors	22	64 ± 4	11 (50.0%)	1.488 ± 0.308	
Zhao et al., 2021 [43]	China	Non-severe	112	61.3 ± 2.8	45 (40.2%)	1.325 ± 0.19	8
		Severe	60	70.6 ± 11.6	37 (61.7%)	1.075 ± 0.053	
Zhou et al., 2022 [44]	China	Non-severe	126	44.95 ± 4	40 (31.7%)	0.808 ± 0.038	8
		Severe	52	54.8 ± 4.2	32 (61.5%)	0.773 ± 0.032	

Legend: AKI: acute kidney injury; NS: not specified.

## Data Availability

The data that support the findings of this study are available on request from the corresponding author (L.S.).
